# Retraction: Design and performance test on segmented‐differential threshing and separating unit for head‐feed combine harvester

**DOI:** 10.1002/fsn3.3719

**Published:** 2023-10-18

## Abstract

Tian, L, Lin, X, Xiong, Y, Ding, Z. “Design and performance test on segmented‐differential threshing and separating unit for head‐feed combine harvester.” *Food Sci Nutr.* 2021; 9: 2531–2540 (https://doi.org/10.1002/fsn3.2202).

The above article, published online on March 8, 2021 in Wiley Online Library (wileyonlinelibrary.com), has been retracted by agreement between the authors, the journal Editor in Chief, Y. Martin Lo, and Wiley Periodicals LLC. The retraction has been agreed as the author has discovered an incorrect coded value in the levels of test factors in Table 2, thus affecting the interpretation of the results so that the conclusions of this manuscript are not reliable.

Tian, L, Lin, X, Xiong, Y, Ding, Z. “Design and performance test on segmented‐differential threshing and separating unit for head‐feed combine harvester.” *Food Sci Nutr.* 2021; 9: 2531–2540 (https://doi.org/10.1002/fsn3.2202).

The above article, published online on March 8, 2021 in Wiley Online Library (wileyonlinelibrary.com), has been retracted by agreement between the authors, the journal Editor in Chief, Y. Martin Lo, and Wiley Periodicals LLC. The retraction has been agreed as the author has discovered an incorrect coded value in the levels of test factors in Table 2, thus affecting the interpretation of the results so that the conclusions of this manuscript are not reliable.

